# Modelling the Transport of Nanoparticles under Blood Flow using an Agent-based Approach

**DOI:** 10.1038/srep10649

**Published:** 2015-06-10

**Authors:** Gavin Fullstone, Jonathan Wood, Mike Holcombe, Giuseppe Battaglia

**Affiliations:** 1 Department of Chemistry, University College London, UK; 2 MRC Centre for Molecular Virology, University College London, UK; 3 Sheffield Institute for Translational Neuroscience, University of Sheffield, UK; 4 Department of Computer Science, University of Sheffield, UK

## Abstract

Blood-mediated nanoparticle delivery is a new and growing field in the development of therapeutics and diagnostics. Nanoparticle properties such as size, shape and surface chemistry can be controlled to improve their performance in biological systems. This enables modulation of immune system interactions, blood clearance profile and interaction with target cells, thereby aiding effective delivery of cargo within cells or tissues. Their ability to target and enter tissues from the blood is highly dependent on their behaviour under blood flow. Here we have produced an agent-based model of nanoparticle behaviour under blood flow in capillaries. We demonstrate that red blood cells are highly important for effective nanoparticle distribution within capillaries. Furthermore, we use this model to demonstrate how nanoparticle size can selectively target tumour tissue over normal tissue. We demonstrate that the polydispersity of nanoparticle populations is an important consideration in achieving optimal specificity and to avoid off-target effects. In future this model could be used for informing new nanoparticle design and to predict general and specific uptake properties under blood flow.

The blood circulatory system is a dense network of blood vessels that acts to carry vital nutrients and signals to the tissues of the body, whilst simultaneously removing waste products. The circulatory system is involved in the exchange of gases, ions, macromolecules and even cells between the blood and tissue. The ability of the blood to distribute variable payloads to tissues is highly relevant to drug delivery^1^. Recent work has focused on utilising nanoparticle-based drug-carrier systems to aid retention and specific delivery of a multitude of potential therapeutics to hard-to-access tissues, such as tumours and the central nervous system (CNS). Nanoparticles is the general term for a diverse group of nanoscale particles designed and modified to improve desirable properties such as immune system evasion, tissue penetration, cellular uptake, cellular trafficking and cargo-delivery^2^,^3^. Their composition can vary greatly, giving different surface chemistries, shapes and sizes. A number of approaches have been taken to better understand how such properties of nanoparticles affect their applicability as a drug-delivery system. These include classic biological studies using *in vitro* and *in vivo* systems as well as *in silico* approaches^4^,^5^,^6^.

The use of *in silico* methodology has opened new avenues in the field of drug discovery and development, including in the design of novel chemical inhibitors by protein structural analysis^7^,^8^, drug library screening using quantitative structure-activity relationship (QSAR) methods^9^,^10^ and multi-scale modelling^11^. Computational fluid dynamics (CFD) is a well known *in silico* approach used throughout the fields of engineering, physics and increasingly biology. It aims to use computational methods to resolve the Navier-Stokes equations governing fluid flow, under set conditions and geometry. The Navier-Stokes equations refer to three coupled partial differential equations (PDEs) with the vector equation for the conservation of momentum being
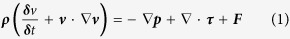
where***ρ*** is the density, ***δt***is the time step, 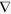 is the del operator, 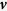 is the velocity, 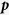 is the pressure, 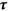 is the total stress tensor and ***F*** is the force. Note: All variables and typical values are included in Supplementary Table 1.

The continuity equation describing the conservation of mass can be expressed as:
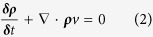
while the equation for conservation of energy is
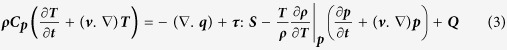
where ***C***_***ρ***_ is the specific heat capacity at constant pressure, ***T*** is the absolute temperature, ***q*** is the heat flux vector, ***S*** is the rate of strain tensor and ***Q*** is the heat sources. CFD models are obtained by finding a solution to the momentum equation (1), which simultaneously satisfies equations (2) and (3). Only in a few cases do absolute solutions exist and are limited to conditions where non-linear parameters are equal to 0, for example Poiseuille’s flow or Stokes’ creeping flow. In more complex flows, computational methods to obtain solutions that correlate well with experimental data have been developed. These methods include finite volume, finite difference and finite element methods. Finite element methods in particular have a history of providing robust solutions over a range of flows. They aim to obtain the equation of momentum in a ‘weak form,’ that can be iteratively assessed at mesh points, thus giving an effective model of flow^12^.

Many studies have utilised CFD methods to study the behaviour of the blood, predominantly focusing on cardiovascular diseases in larger vessels^13^,^14^. Much less attention has been paid to the behaviour of the blood in capillaries and its role in effective delivery to tissues. The capillary is the smallest of the blood vessels, ranging from 5 to 40 μm in diameter giving it a characteristic high surface area to volume ratio, maximising the potential for blood-tissue exchange. Furthermore, the vessel walls of capillaries consist of an endothelial cell layer, just one-cell thick, thereby minimising transport times across the vessel wall. Blood flow through capillaries is driven by the pressure difference between the pre-capillary pressurised arterioles and the post-capillary low-pressure venules. Capillary flow is laminar, as defined by the Reynold’s number (Re), the ratio between viscous and inertial forces:
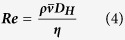
where 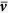 is the mean velocity and ***D***_***H***_ is the hydraulic diameter. Capillaries have a Reynold’s number of 0.001, well below the range of transient flow of ~2400, due to their small length scales and high viscosity. Laminar flow is characterised by flow in parallel layers with no mixing. Flow at the vessel wall is vastly reduced due to friction with the stationary vessel wall, with the velocity increasing towards the centre of the vessel in a parabolic manner. In addition to laminar forces, other forces also act upon nanoparticles within the blood. In particular, thermal fluctuations, also known as Brownian motion, causes random movement of particles in relation due to the thermal energy of the system. The displacement of particles is related to the diffusion coefficient (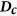) calculated from the thermal energy of the system and the Stokes drag of the particles by the Stokes-Einstein equation:
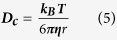
where***k***_***B***_ is the Boltzmann constant and ***r*** is the particle radius.

At the vessel surface, nanoparticles can interact and traverse the endothelial layer in numerous ways. In many tissues, 60 nm pores, called fenestrations, punctuate the microvasculature, which allows free exchange of many substrates. However these fenestrations can be considerably larger in vessels supplying tumours, with fenestrations up to 400 nm^15^. The endothelial cells themselves can also regulate the exchange of numerous substrates between blood and tissue by receptor-mediated transport mechanisms and cellular trafficking.

Blood, as a fluid, has several properties that must be considered when utilising CFD methods for modelling. Most of these properties are a consequence of the high density of cells and cell fragments within the blood including red blood cells, white blood cells and platelets. Red blood cells (RBCs) are the most numerate blood cell, constituting 38-46% of the volume of blood, a measure referred to as the haematocrit. White blood cells and platelets constitute about 1% and <0.5% of blood volume respectively. In capillaries, the haematocrit is reduced to 10-12%, reflecting the hydrostatic pressure^16^. In its resting form, the red blood cell is a biconcave disk measuring ~7.8 μm in diameter, comparable to the size of capillaries. However, RBCs deform under shear stress into numerous conformations favourable for reduced resistance to flow in restricted geometries^17^,^18^. The presence of RBCs creates complications in obtaining accurate solutions to Navier-Stokes equations. RBCs confer non-Newtonian properties to the fluid. This means that the viscosity will alter under shear stress in a non-predictable way. This can be explained by the dependence of viscosity on shear-rate, haematocrit, red blood cell conformation, red blood cell aggregation and plasma viscosity^19^,^20^. These properties are also simultaneously related to the viscosity, therefore making complete solutions very difficult to obtain.

Agent-based modelling is a type of modelling specifically designed for systems analysis. It deconstructs a system into components, known as agents, and assigns mathematical expressions to describe their behaviour. Subsequently, by combining the different agents together in simulations, through agent-agent and agent-environment interactions, emerging systems behaviour can be observed. We here utilise FLAME (Flexible Large-scale Agent-based Modelling Environment), a generalised agent-based modelling framework for flexible modelling whilst simultaneously maintaining parallelisation optimisation (for more details on agent-based modelling and parallelisation with FLAME, see Supplementary information).

Our interest in nanoparticle delivery has prompted us to further explore the role of fluid dynamics in the distribution of nanoparticles within capillaries and their subsequent uptake across the vessel walls. Here we describe the building of an agent-based model using the FLAME framework, which models the behaviour of nanoparticles at the capillary level in the presence of RBCs. We demonstrate the use of this model by illustrating the role that RBCs play in improving the distribution of nanoparticles within the vessel. Furthermore, we establish the use of this model in aiding nanoparticle design by showing how nanoparticle size and polydispersity are important considerations in conferring selectivity for tumour delivery.

## Results and Discussion

### Building an Agent-based Model of Blood Flow

Agent-based modelling has previously proven to be a powerful tool for predictive biological modelling. Its integration with more classic CFD approaches allows great potential for testing the blood flow dependent behaviour of nanoparticles. Blood flow dynamics in capillaries are integral for distribution and subsequent uptake of blood-borne molecules. We aimed to create a simple core model that simulates blood flow with the intention of studying a variety of other processes, including but not limited to, nanoparticle distribution studies, receptor binding dynamics, the effect of ligand density, and cellular trafficking, plus the effect of varying flow conditions on all of the aforementioned. A summary of this is provided in the schematic in Supplementary Figure 2.

In the microvasculature, where blood cell and capillary diameter are comparable, blood cells severely influence the fluid dynamics of blood flow. Whilst white blood cells and platelets are present in the microvasculture, the reduced number of both cells compared to red blood cells and the small size of platelets limits the influence of these cells. Therefore, we concentrate on red blood cells as the major driver of fluid dynamical changes in the microvasculature. Whilst inclusion of RBCs is important to flow modelling, direct modelling of red blood cell behaviours, such as deformation and aggregation, has been largely omitted. Previous studies have utilised several techniques, most notably immersed finite element methods, to incorporate red blood cell deformation and red blood cell interactions within flow models^21^,^22^,^23^. Although a similar approach could be utilised within the model described, the additional computational burden required would limit the capabilities of the model to include much of the desired additional functionality. In these theoretical studies and experimental observations, a number of red blood cell conformations have been described, including, the slipper-like, the parachute, bullet-like and disk conformations. A number of these papers also produced phase-diagrams relating the conformation to other properties of the flow, such as shear rate, confinement and flow velocity^22^,^23^. The slipper phase, according to the work of Fedosov and colleagues^22^,^23^, occurs when both shear rates and confinement are low, however the shear rates and confinement parameters of our simulations favour the parachute conformation^23^.

**Figure 1:**
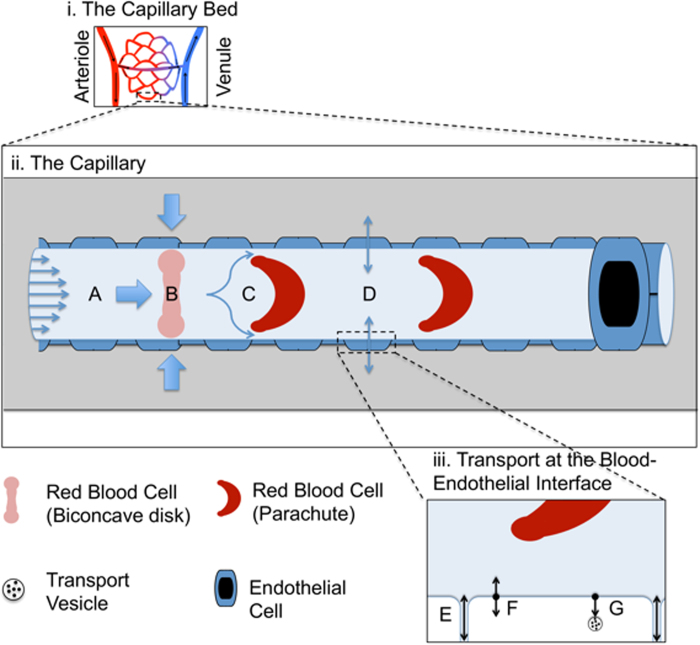
Model Summary Schematic. The model focuses on a single capillary (ii) taken from the capillary bed (i). The model includes the effect of laminar flow (**A**) on red blood cells in their native biconcave disk shape (**B**) causing the deformation of red blood cells into a parachute conformation (**C**). This subsequently affects distribution and fluid dynamics of particles in the fluid-phase of the blood, which will in turn affect interactions at the vessel wall (**D**). At the interface of blood and vessel wall (iii), particles proximal to the vessel wall can pass through cellular junctions between cells or fenestrations within cells and therefore freely exchange with the interstitial fluid according to the diffusion gradient (**E**). Particles may bind corresponding proximal receptors on the endothelial cells (**F**) they may then be released or internalised and subjected to various cellular trafficking systems (**G**).

Several studies have demonstrated that both specific and non-specific interaction with proteins can affect the nanoparticle residency in the blood^24^,^25^. Protein interactions are dictated by the surface chemistry and charge. A common solution to this is to use a coating that limits protein adsorption such as polyethylene glycol (PEG) or similar^26^. This generally improves the systemic half-life and reduces immune cell interaction properties, thus increasing the potential therapeutic load at target tissues^27^,^28^. Therefore, in our simulations we can assume that such nanoparticles will be inert. In previous studies, it has been demonstrated that certain compositions of nanoparticles can interact with both themselves and red blood cells, with varying effects on distribution and cellular interactions^29^. However, these properties are likely to be individual to the nanoparticle formulation used and therefore have been omitted from our model, except for a simple rule that neither can occupy the same space. However, specific interactions are easily implementable within the model at a later stage if appropriate. A diagrammatic summary of the model built is provided in Fig. 1.

### Brownian Forces become the Dominant Force at the Vessel Wall Interface

**Figure 2:**
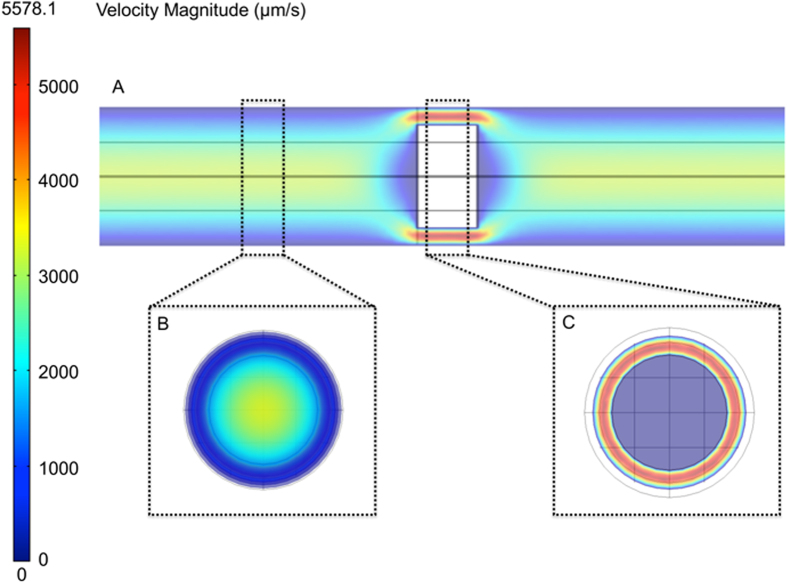
Heat Mapping of Laminar Forces. Longitudinal cross-section (**A**) demonstrating velocity of blood flow along the vessel, with subsequent latitudinal cross-sections proximal to the red blood (**B**) cell and distal to the red blood cell (**C**).

**Figure 3:**
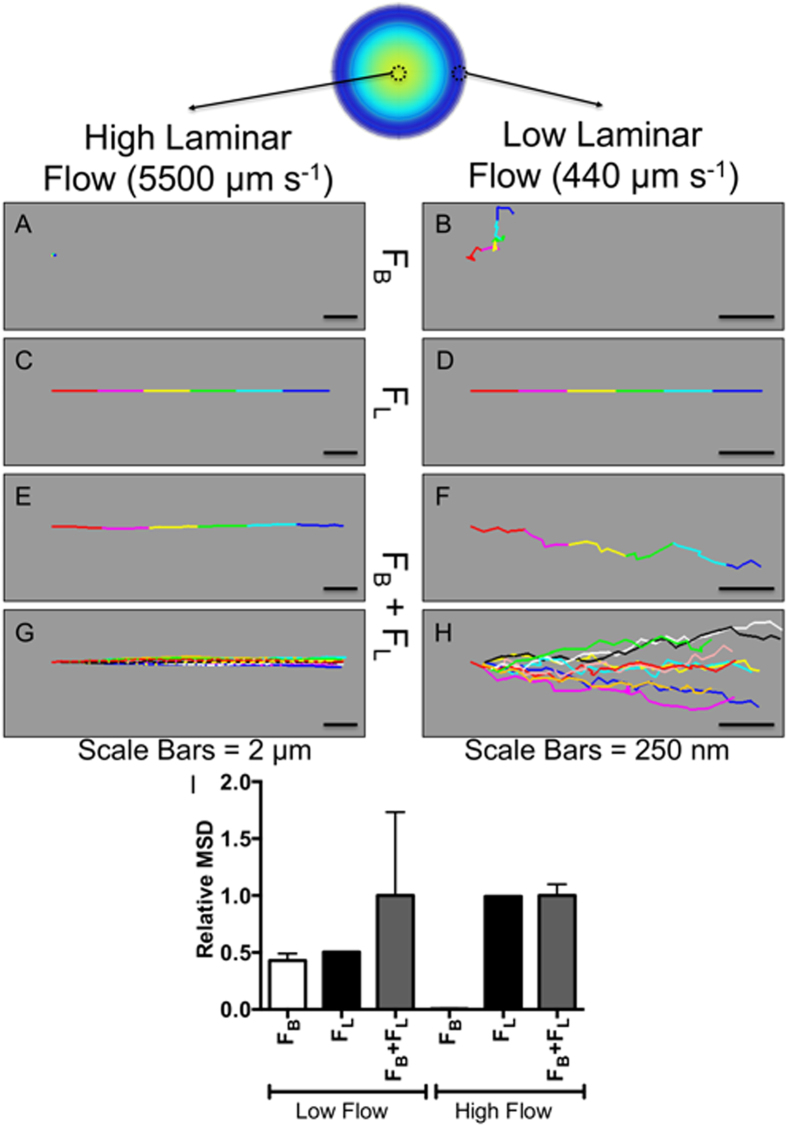
Balance of Brownian to Laminar Forces. 100 nm nanoparticle traces under only Brownian motion (**A** and **B**), only laminar flow (**C** and **D**) and both Brownian and laminar forces (**E** and **F** [single nanoparticle], **G** and **H** [ten nanoparticles]) at the edge and centre of the vessel. The relative contribution of laminar (F_L_) and Brownian (F_B_) forces to the mean squared displacement at low laminar and high laminar force is demonstrated (**I**). (Time = 0.003 s, ***δt*** = 0.0001 s).

To validate the correct operation of the finite element method used, heat maps were produced of flow velocity in longitudinal (Fig. 2A) and latitudinal (Fig. 2B,C) vessel slices at a physiologically relevant haematocrit of 10.7%. The heat maps demonstrate a clear relationship between proximity to the vessel wall and a decrease in flow velocity (Fig. 2B). In regions containing RBCs, flow velocity is increased around the cells as they move at slower rates than the fluid phase (Fig. 2C). Next, a comparison of the various influences of Brownian motion and laminar flow on final nanoparticle movement was made in different regions of the vessel. Typical nanoparticle traces for Brownian motion (Figs. 3A,3B) alone, laminar flow alone (Figs. 3C,3D) and laminar flow with Brownian motion (Fig. 3E-H) are shown for a 100 nm nanoparticle at the centre of the vessel and the edge of the vessel. At the centre of the vessel, laminar forces are expected to be high (*ν* = 5500 *μm* *s*^−1^) as it is furthest away from the non-slip boundary. The relative impact of Brownian forces (Fig. 3A) is therefore consequently very small, relative to the laminar forces (Fig. 3C), leading to only minor fluctuations from laminar flow (Figs. 3E,3G). However at the vessel wall (Fig. 3D), the flow velocity is vastly reduced (*ν* = 440 *μm* *s*^−1^), thus making Brownian forces more significant to the displacement of the particle (Figs. 3B,3F,3H). This is summarised by the relative contributions of Brownian and laminar forces to the total mean squared displacements (MSD) of nanoparticles at low and high shear flow (Fig. 3I). At low laminar forces, Brownian forces contribute almost equally to laminar forces in the displacement of the particle, where as the relative contribution of Brownian forces at high laminar forces is negligible. The dominance of Brownian forces and reduction in laminar forces allows maximum contact time between potential substrates and the vessel wall interface. It also allows nanoparticles proximal to the vessel wall but not within binding range to diffuse closer to the vessel wall, thereby permitting binding. Although not included in our current model, the significance of Brownian forces at the vessel wall is likely to be further increased by the presence of a gel-like layer at the cell wall called the glycocalyx. This layer consists of glycosylated proteins (glycoproteins) and lipids (glycolipids) that can project 200-500 nm within the vessel. It can also regulate accessibility of various blood components to the wall based on particle properties such as charge and size^30^.

### Red Blood Cells enhance Nanoparticle Dispersion

**Figure 4:**
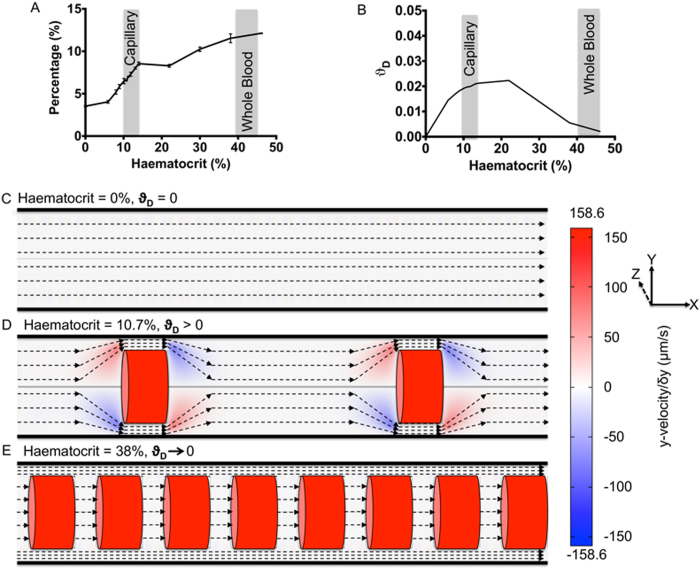
The Effect of Haematocrit on Nanoparticle Distribution and Motion. The increased dispersion of nanoparticles to the vessel edge as a function of increasing haematocrit is demonstrated by the percentage of nanoparticles within 20 nm of the vessel wall (**A**). The average dispersion factor is then shown as a function of haematocrit (**B**). Typical ranges for capillary and whole blood haematocrits are indicated. Streamlines of different types of flow observed and heatmaps of the velocity in the y-plane are shown with respect to haematocrit, at no haematocrit (***H*****%** = 0) a one phase Poiseuille’s flow (**C**), at 10.7% representative of intermediate haematocrits (0 < ***H*****%** < 20) an interacting flow between cellular and cell-free layers (**D**) and a haematocrit of 38% representative of high haematocrits (***H*****%** > 20) separate cellular and cell-free layers (**E**).

In order to see the effect that RBCs have on flow dynamics and subsequent nanoparticle dispersion, the behaviour of nanoparticles at varying haematocrits was studied. State and process order graphs for the parallel execution of these simulations are provided in Supplementary Figure 3. The haematocrit range used was from acellular (0%) to whole blood (45%), including a high density of simulations at the physiologically relevant range for a capillary (10-12%). The viscosity used in resolving the Navier-Stokes equations was related to the haematocrit in order to account for the non-linear increase in viscosity usually observed with higher haematocrit (Supplementary Figure 4). Supplementary Figure 5A shows the average position of a nanoparticle relative to the centre of a 4000 nm vessel, as a function of haematocrit. At haematocrits of 0%, an even dispersion of nanoparticles would be expected, giving a theoretical average of ~2700-2800 nm with a nanoparticle diameter of 100 nm. However, it would be expected that this value would increase due to volume exclusion with increasing amount of RBCs located towards the centre of the vessel, as observed in Supplementary Figure 5A. Furthermore, to correlate this with likelihood of increased delivery, the percentage of all nanoparticles that would be within a binding range of the vessel wall (Fig. 4A), given as 20 nm from the proximal edge of the nanoparticle to the vessel wall, was quantified. A marked increase is observed with increasing haematocrit, particularly across the physiologically relevant range, further demonstrating that RBCs aid the dispersion of nanoparticles to the vessel wall. The increase in haematocrit also had an effect on the average velocity of nanoparticles across the whole vessel (Supplementary Figure 5B) and for nanoparticles within 20 nm of the vessel wall (Supplementary Figure 5C). It can be expected that this would influence both the motion of particles at the vessel wall, as it alters the laminar-Brownian force ratio and their subsequent transport at the vessel surface, due to increased wall shear stress.

Similar conclusions, demonstrating improved nanoparticle distribution with higher haematocrit, were made by Tan and colleagues^31^. Whilst their conclusions are similar, they focus on scenarios in the presence or absence of RBCs, using a haematocrit of 38%, which is considerably higher than the 10-12% expected in capillaries. Therefore, it is unclear from this work whether this observation would be as significant in more physiological conditions. Furthermore, the vessels used in their study were bigger than that described here (20 μm and 11 μm compared to 8 μm), making immediate comparison of data difficult.

To further study the flow dynamics with differing haematocrits, an average dispersion factor was calculated for vessels with varying haematocrit. The dispersion factor is the ratio between radial velocity (towards the vessel edge) and the longitudinal velocity (towards the end of the vessel) with the Radial Velocity (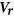) being.
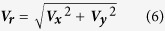
where 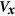 is the velocity in the x-direction, 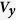 is the velocity in the y-direction. We can thus define the Dispersion Factor (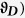 as
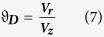
where 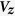 is the velocity in the z-direction, similarly we can define the Average Dispersion Factor (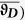 as
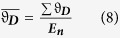
where 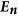 is the number of elements.

A dispersion factor of 0 would indicate that flow is parallel to the vessel wall, giving no dispersion, where as, any increase in dispersion factor would show trajectories away from parallel to the vessel wall. Figure 4B demonstrates an initial increase in dispersion factor as a function of haematocrit. However, above haematocrits of 20-25%, this then falls and tends back towards 0. This would indicate that an intermediate range of haematocrit could be better for allowing maximum dispersion of nanoparticles towards the edge of the vessel. To understand why the average dispersion factor shows increased dispersion with lower haematocrits to higher, further observation of flow dynamics was performed using heat mapping of the dispersion factor at individual elements. Figures 4C,4D,4E are schematics of the three dominant flow streamlines observed throughout the simulations imposed on heatmaps of the velocity in the y-axis (perpendicular to the vessel). In Fig. 4C, in the absence of RBCs, flow can be considered to be a Poiseuille’s flow and will result in an expected and observed average dispersion factor of 0. Figure 4D demonstrates that flow, in the presence of an intermediate haematocrit, will manoeuvre around the RBCs and will lead to the observed increase in average dispersion factor. However, at higher haematocrits, the space between RBCs is significantly reduced, and as such, two separate phases are formed (Fig. 4E). A cellular-phase is formed where, due to the reduced space between RBCs, flow between RBCs occurs at the same velocity as that of the RBCs. A separate cell-free phase is created at the edge of the vessel; the flow of this phase occurs such that the cellular-phase resembles flow past a cylinder moving at a rate equal to the red blood cell velocity. Interaction between the two phases becomes minimal as the space between RBCs is reduced, consequently resulting in the decline of average dispersion factor observed with increasing haematocrit above 20-25% in Fig. 4B. A similar but less defined pattern in flow is also apparent in the work of Tan and co-workers and McWhirter and colleagues^18^,^31^.

The consequence of a low average dispersion factor is that only nanoparticles already proximal to the vessel edge can be transported across the vessel wall. However with higher average dispersion factors, nanoparticles at the centre of the vessel will be transiently dispersed from the centre to the vessel edge and vice versa with flow. This permits a greater number of nanoparticles the opportunity to be transported at the vessel wall.

### Nanoparticle Size Selectively Targets Delivery to Tumour Tissues

**Figure 5:**
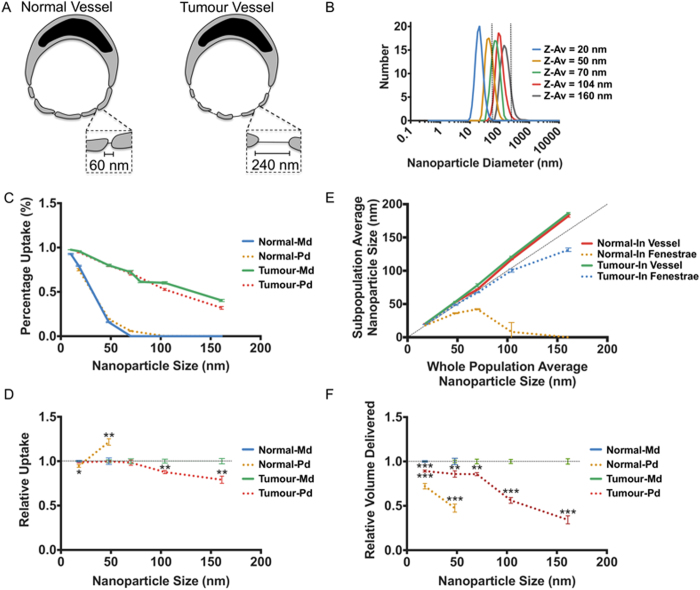
Specific Nanoparticle Targeting of Tumour Tissue. The delivery across fenestrations was accessed for normal fenestration sizes and larger fenestrations resembling vasculature of cancer tissue (**A**). Polydisperse (Pd) nanoparticle populations generated from DLS size data were used alongside monodisperse (Md) populations of the average sizes, fenestration sizes are indicated by dashed lines (**B**). The percentage uptake after 0.5 s is compared between normal fenestrations and tumour fenestrations (**C**). Polydisperse populations are compared to their sized matched monodisperse populations for uptake in fenestrations at 0.5 s; a dashed baseline is used in place of the normalised monodisperse populations but error bars are included used (**D**). A comparison was then made between the average size of the nanoparticles within fenestrations and those within the vessel for the polydisperse populations (**E**). Finally the relative volume of nanoparticle mass delivered across fenestrations was compared between polydisperse populations and their equivalent monodisperse population at 0.5 s; a dashed baseline is used in place of the normalised monodisperse populations but error bars are included used (**F**). * P < 0.01, ** P < 0.005, *** P < 0.001.

A key factor in nanoparticle efficacy is their ability to selectively target the tissue of interest, whilst avoiding potential off-target effects at other tissues. In order to demonstrate the capability to increase specificity of nanoparticles for tumours, the ability of different sized nanoparticles to traverse fenestrations with pore size corresponding to both normal vessels (60 nm) and those associated with tumours (240 nm), was tested (Fig. 5A). The core model described previously was utilised using a haematocrit of 10.7%, with the inclusion of a fenestration agent and function to add fenestrations to the vessel wall. The relevant state and process order graphs, generated by FLAME, for this model is included in Supplementary Figure 6.

Nanoparticle sizes used were 10 nm, 20 nm, 50 nm, 70 nm, 80 nm, 100 nm and 160 nm. Polydisperse samples with an average diameter corresponding to 20 nm, 50 nm, 70 nm, 100 nm and 160 nm (Fig. 5B) were also trialled to compare data with that expected from corresponding biological experiments. These simulated polydisperse populations were generated to correspond to nanoparticle populations measured using dynamic light scattering (DLS) analysis (Supplementary Figure 7). Figure 5C demonstrates that across all size ranges, delivery across tumour vessel fenestrations is vastly increased compared to normal capillaries. Furthermore efficient delivery is still achieved with nanoparticles at 50 nm, 70 nm, 80 nm and 100 nm, where little or no delivery is achieved in normal fenestrations, suggesting that this size range can improve specific delivery to tumour tissue. This concept forms part of the enhanced permeability and retention (EPR) effect or passive-targeting, that has been proven previously in many studies^32^,^33^. The EPR effect is based on the combination of the increased permeability of the vasculature supplying tumours and reduction or absence of lymphatic vessels that form a drainage network from tissues back to the blood. Therefore nanoparticles accumulate more readily in these regions and are not cleared as effectively as other tissues given a prolonged therapeutic response. In these studies an important feature was the half-life of the nanoparticle, which is related to size, shape and surface chemistry. Generally sizes between 40-100 nm have previously been demonstrated to persist in the blood due to reduced loss due to blood extravasation and filtration by the reticuloendothelial systems of the liver, spleen and kidneys^15^,^34^,^35^. Our results for specific delivery to a tumour fall within this range, thus making the data more significant with respect to translation from simulation to experimental data. Nanoparticle mechanical properties will also influence the EPR effect. Flexible nanoparticles, such as liposomes and polymersomes, have been demonstrated to translocate across pores considerably smaller than themselves^36^,^37^. Therefore, flexible nanoparticles may perform differently than more rigid nanoparticles, such as gold. The addition of active targeting domains, such as iRGD, to nanoparticles can also improve uptake properties of nanoparticles from the blood^38^,^39^. It should be noted that whilst irregular and larger fenestrations have been reported at tumour vasculature, fenestration density, irregular flow and poor cellular junction formation also are characteristic of tumour vasculature, inevitably influencing overall uptake into tumour tissues^35^. This experiment also demonstrates the importance of the inclusion of Brownian fluctuations within laminar flow models, as no delivery could be achieved without its consideration.

Whilst a similar effect was demonstrated using both monodisperse and polydisperse populations, delivery using 100 nm and 160 nm populations was significantly (P < 0.01) reduced in the polydisperse samples (Fig. 5D), likely reflecting the presence of a minority of nanoparticles of greater size than the tumour vessel fenestrations. In parallel, in polydisperse populations corresponding to 70 nm, a small but critical increase is observed in the delivery to normal tissue (P = 0.00007), reflecting presence of particles below the 60 nm cut off. Therefore, polydispersity is a key consideration in the design of nanoparticles for improved specificity using the EPR effect. In order to measure the effect of size and polydispersity on specificity for tumour tissue, we measured the ratio of nanoparticles delivered to tumours against those delivered to normal tissue:
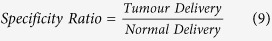


Furthermore, to establish a measure that accounts for both the specificity and the overall delivery efficiency we also calculated a value we refer to as the specificity score and is calculated as:
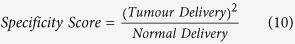


Supplementary Figure 8 demonstrates the change in specificity ratio and score with increasing nanoparticle size. The specificity ratio increases dramatically from 80 nm and above, reflecting the low delivery into normal tissue for larger size nanoparticles. The most efficient size for obtaining specificity is at 160 nm, reflecting the fact that very few nanoparticles in the population will be able to successfully translocate across 60 nm fenestrations. However, whilst it has high specificity, it has relatively low delivery efficiency to the tumour tissues. It is expected that the amount of nanoparticles in the bloodstream will decline over time due to blood clearance routes such as the reticuloendothelial system. Therefore, the 100 nm sample may be more suitable for optimal delivery to provide only marginally reduced specificity (1.08 fold decrease) but greater delivery efficiency (1.65 fold increase). This is reflected in the greater specificity score observed for 100 nm compared to 160 nm in Supplementary Figure 8.

In the analysis of polydisperse nanoparticle uptake, we observed a marked difference in size between those entering fenestrations and those in the vessel (Fig. 5E), with smaller sizes being taken up more readily as would be expected from the previous data. To quantify a potential effect of this on cargo delivery we then compared the volume of nanoparticle delivered between the polydisperse samples and their respective monodisperse samples (Fig. 5F), based on the assumption that the volume of a nanoparticle is directly related to the amount of cargo it can carry. Across all samples, the polydisperse populations delivered significantly less volume across the fenestrations (as much as 66% reduction for the 160 nm population). This demonstrates that monodisperse nanoparticle populations, often used in computational simulations, may further give poor correlation with observed experimental results, where delivery quantification will be directly related to volume of nanoparticle itself or the related volume of cargo. Whilst we consider here the effects of polydispersity and size for a specific scenario in nanoparticle delivery, size and therefore polydispersity also influence general nanoparticle properties such as immune system interaction, clearance, tissue penetration and diffusion rates.

## Conclusions

In conclusion we have successfully implemented an agent-based model of nanoparticle behaviour under physiological blood flow. This model has been designed to allow the facile inclusion of cellular interactions and trafficking. This model has given us insight into the integral role of RBCs in the distribution of nanoparticles to the vessel walls. We have used this model to demonstrate that both size and polydispersity of nanoparticles must be considered together when targeting tumour tissue using the EPR effect. We predict that in future this model will be used to aid nanoparticle design to further improve tumour delivery and specific delivery to other clinically important tissues such as the CNS.

## Methods

### Agent-based Modelling

All models were written in FLAME and executed, in parallel, on the Iceberg High Performance Computing Cluster based at the University of Sheffield (http://www.shef.ac.uk/wrgrid/iceberg). The state and process order graphs presented in Supplementary Figures 3 and 6 are automatically produced by FLAME during compilation in the DOT language. These can be visualised using the open source Graphviz package (http://www.graphviz.org). Our simulations concentrate on the singular capillary, which is treated as a hollow cylinder, with no slip boundaries. Although capillary diameters vary, we have concentrated our simulations to vessels around 8-12 μm in diameter, although the model is feasibly extendible beyond these limits. The model is constructed combining the following agents:

### Red Blood Cells

The number of RBCs is generally confined to give a physiologically accurate haematocrit of 10-12%. RBCs are maintained in an orientation perpendicular to the flow and at the centre of the vessel. RBCs are set to move at a rate determined by the pressure and viscosity; their effective radius, after deformation is taken from the literature^16^.

### CFD Methods

In order to simplify the solutions to the Navier-Stokes equations, RBCs are treated as separate objects that influence flow by creating transient moving wall boundaries. A finite element approach, using Galerkin/least squares (GLS) methods, is then used within this geometry to aid generation of a solution to the Navier-Stokes problem for the fluid-phase^12^. Acellular blood (plasma) is a Newtonian fluid and therefore we have treated the fluid flow as Newtonian. Blood flow is treated as incompressible, thus meaning the density of the fluid remains unchanged, such that:
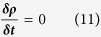


Therefore, by substituting equation (11) into (1) and (2) we achieve the incompressible Newtonian form for the conservation of momentum (12) and incompressible form of the continuity equation (13):
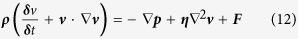

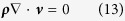
where 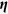 is the dynamic viscosity and 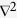 is the Vector Laplacian.

Equation (11) also leads to decoupling of the energy equation (3) from (12) and (13). Newton’s second law can then give Nanoparticle motion as
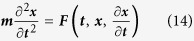
where ***m*** is the mass, 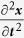 is the acceleration in x, ***t*** the time and ***x*** the x-coordinate. Brownian motion forces can be simulated by the methods presented by Andrews and Bray^40^, requiring the calculation of the translational diffusion coefficient (***D***_***c***_), using the Stokes-Einstein equation:
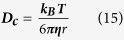
where***k***_***B***_ is the Boltzmann constant and ***r*** is the particle radius. The calculation of the standard deviation (***σ***) for a Gaussian distribution from the diffusion coefficient:
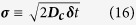


Random numbers can then be generated to fit the resulting Gaussian using the modified Box-Muller transformation^41^,^42^:
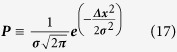


***P***is the probability of a change in x-coordinate of value ***Δx***. The generation of these random numbers in all 3 axes gives a force vector that simulates the random walk (***F***_***B***_). Which can be added to force vectors generated from the Navier-Stokes (***F***_***L***_) to obtain total force (***F***_***Total***_) acting upon the nanoparticle:
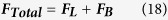


The vessel wall is treated as a reflective boundary in the event of a nanoparticle-vessel wall collision.

### Nanoparticles

Nanoparticles are modelled as spheres with variable size. They can be modelled to be of a ubiquitous size or to fit a range given by nanoparticle size analytical techniques, including DLS and Nanosight^®^ tracking analysis (NTA). Nanoparticle-nanoparticle and nanoparticle-RBC interactions are neglected with the exception that neither can occupy the same space.

DLS distribution curves are generated by the sorting of particle size into size classes by the Zetasizer^®^ software package. The simulated nanoparticle agents are assigned a size class based on probabilities calculated from the cumulative frequency data. The absolute size is then assigned by weighting the probability of sizes within the class by the neighbouring classes. Supplementary Figure 7 demonstrates distributions of different sized polydisperse nanoparticles from DLS analysis (Supplementary Figure 7A) and their respective simulated distributions (Supplementary Figure 7B).

### Fenestrations

Fenestrations are modelled as narrow pores perpendicular to the vessel wall. Fenestration diameter is maintained at 60 nm (typical for a normal vessel) or 240 nm (tumour vessel). Their density is obtained from the literature^43^. Nanoparticle movement within pores is generated through Brownian motion, using a shorter time-step than in the main vessel, due to the narrow geometry.

### Mean Squared Displacement

The MSD of a nanoparticle at time 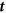 is calculated by:
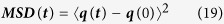
where 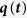 is the position of the particle at time 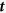 and 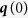 is the position of the particle at time 0.

### Statistical Analysis

P-values were obtained and statistical significance determined using multiple t-tests by the Holm-Sidak method^44^.

## Additional Information

**How to cite this article**: Fullstone, G. *et al.* Modelling the Transport of Nanoparticles under Blood Flow using an Agent-based Approach. *Sci. Rep.* **5**, 10649; doi: 10.1038/srep10649 (2015).

## Supplementary Material

Supporting Information
