# Supplementary material for: Modelling the Transport of Nanoparticles under Blood Flow using an Agent-based Approach
Source: Sci Rep. 2015 Jun 10;5:10649. doi: 10.1038/srep10649 (PMC4462051; doi:10.1038/srep10649)
Supplement: Supporting Information [file srep10649-s1.pdf]

Supporting information for

Modelling the Transport of Nanoparticles under Blood Flow using an Agent-based Approach

Gavin Fullstone[†,‡,#], Jonathan Wood[§], Mike Holcombe[#], Giuseppe Battaglia*[†,‡]

[†]Department of Chemistry, University College London, UK
[‡]MRC Centre for Molecular Virology, University College London, UK
[§]Sheffield Institute for Translational Neuroscience, University of Sheffield, UK
[#]Department of Computer Science, University of Sheffield, UK

Scalability of Agent-based Simulations

Agent-based simulations, though powerful in terms of predictive capabilities, are often limited in simulation size by the CPU (Central Processing Unit) power and memory availability. This can be addressed by utilising distributed computing, distributing work and memory across several CPUs, commonly referred to as cores. This creates an entirely new problem, how to efficiently access data from agents or functions operating on other nodes. There are several different ways to approach this issue and to optimise this process to achieve maximum 'speed up' of the simulation.

FLAME (Flexible Large-scale Agent-based Modelling Environment) is a generalised agent-based modelling platform. In FLAME, the user defines agents, their respective memory variables, their respective functions to carry out and data access requirements for those functions, from other functions. This is achieved in a basic form using XMML (X-Machine Markup Language). The functions themselves are then coded in a separate function file or files coded in C. FLAME utilises these two user-generated codes to construct an iterative-based executable model in either serial (non-distributed computing) or parallel (distributed computing). In parallel, FLAME uses a scheduler that prioritises work that will generate communications between cores. Whilst the messages are sent in the background, it will then perform all possible work not dependent on those communications, in order to reduce overhead times associated with parallelisation (as demonstrated in *Supplementary Figure 1*). FLAME's approach to parallelisation of agent-based simulations is outlined, in more detail, in the technical report by Chin and colleagues[1]. The use of distinct functions in FLAME also allows the capability to easily add and remove functions to a simulation, whilst still providing the inherent parallelisation optimisation described. This enables the user to systematically include and exclude certain functions and agents, thus giving profound flexibility in simulation to best answer the research question. This is illustrated for the model described in *Supplementary Figure 2*, where optional functions can be included on top of the basic core model functions.

Variable	Description	Symbol	Typical Value

Vessel Radius	Radius of the Capillary	$\boldsymbol{V_r}$	4 µm
Vessel Length	Length of the Capillary	$\boldsymbol{V_l}$	800 µm
RBC Radius	Radius of RBC	$\boldsymbol{RBC_r}$	3.9 µm
RBC Width	Width of RBC	$\boldsymbol{RBC_w}$	2.5 µm
Haematocrit	% RBCs/Blood (v/v)	$\boldsymbol{H_{\%}}$	10-12 %
RBC Number	Number of RBC Agents to Reflect Haematocrit	$\boldsymbol{RBC_n}$	
Nanoparticle Radius	Radius of Nanoparticle	$\boldsymbol{r}$	10-200 nm
Arteriole Pressure	Pressure at Arteriole End of Capillary	$\boldsymbol{p_a}$	1500-3000 Pa
Venule Pressure	Pressure at Venule End of Capillary	$\boldsymbol{p_v}$	1000 Pa
Pressure Difference	Pressure difference from arteriole to venule end of the capillary	$\boldsymbol{p_\Delta}$	500-2000Pa
Dynamic Viscosity		$\boldsymbol{\eta}$	See Supplementary Figure 6
Temperature	Absolute Temperature	$\boldsymbol{T}$	310 K
Boltzmann Constant		$\boldsymbol{k_B}$	1.381E^{-23} m^2 kg s^{-2} K^{-1}
Cartesian Coordinates		$\boldsymbol{x, y, z}$	
Time Step	Time step per iteration	$\boldsymbol{\delta t}$	0.0001 (laminar) and 0.000001 (Brownian motion)
Velocity	Absolute velocity [in x/y/z]	$\boldsymbol{v\ [v_x, v_y, v_z]}$	
Density	Density of Fluid	$\boldsymbol{\rho}$	1060 kg m^{-3}
Stress Sensor	Shear Stress	$\boldsymbol{\tau}$	
Force	Force [Laminar, Brownian]	$\boldsymbol{F[F_L, F_B]}$	
Specific Heat Capacity		$\boldsymbol{C_p}$	
Heat Flux Vector		$\boldsymbol{q}$	
Strain Rate Tensor		$\boldsymbol{S}$	
Heat Sources		$\boldsymbol{Q}$	
Dispersion Factor	Dispersion Factor, Average Dispersion Factor	$\boldsymbol{\vartheta_D, \overline{\vartheta_D}}$	0 to 1
Element Number	Number of Elements for Finite Element Method	$\boldsymbol{E_n}$	
Mass	Nanoparticle Mass	$\boldsymbol{m}$	
Diffusion Coefficient		$\boldsymbol{D_c}$	

Supplementary Table 1: Names, Denotations and Typical Values of Key Values with the Model. Typical values are given, where appropriate, from information available in the literature.

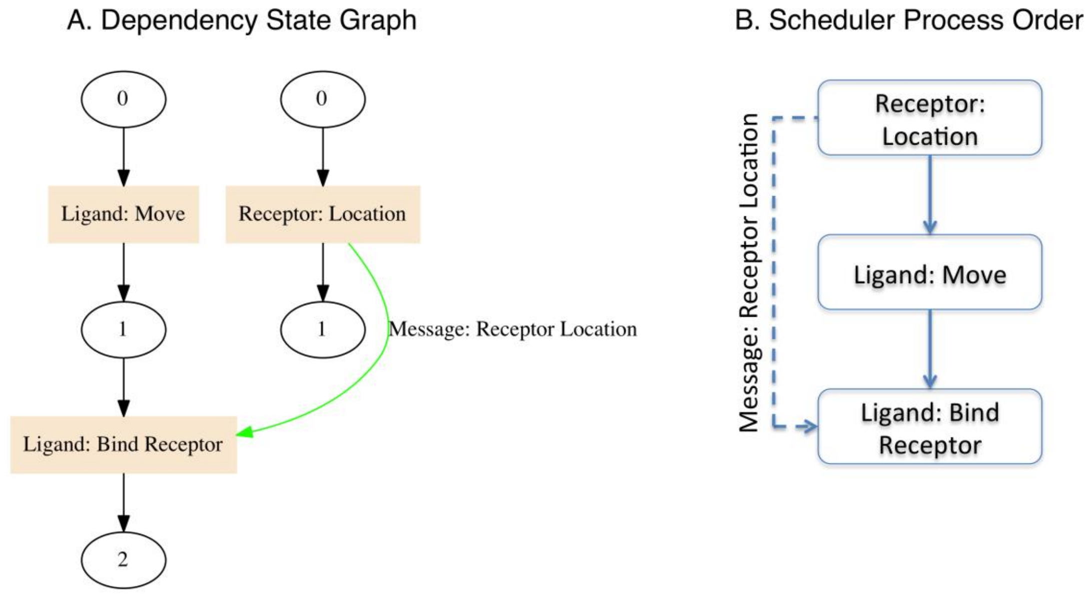

Supplementary Figure 1: Dependency Graph and FLAME Scheduler for a Simple Receptor Binding Model. In the dependency graph (left), 3 functions are displayed, the function 'Ligand: Bind Receptor' is dependent on the carrying out of the preceding function 'Ligand: Move' and also the message 'Receptor Location.' The message is generally limiting to the speed of the process, therefore the scheduler (right) prioritises the function that generates the message, 'Receptor: Location.' Whilst the message is transferring in the background, the scheduler will perform the function 'Ligand: Move' as it is not dependent on the message. This reduces the lag time that the message-dependent function 'Ligand: Bind Receptor' must wait to receive the message before it can use it to perform useful work.

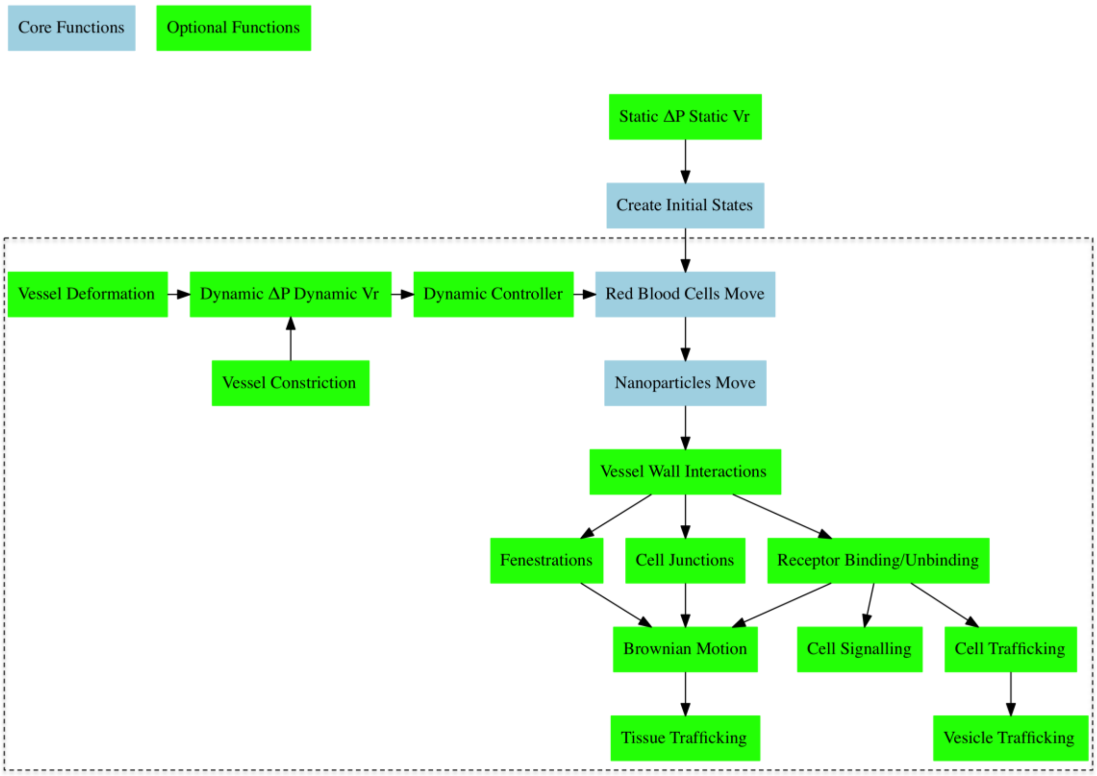

Supplementary Figure 2: Flow Diagram of Model Functions. The flow diagram demonstrates the progression of functions in the model. Core functions that are conserved in all variations of the model are shown in blue, whilst optional functions that can be included in the model, at the user's discretion, are shown in green. The optional functions can be added in a variety of combinations into the core model, to allow flexibility in modelling approach to reflect the experimental question. The functions within the dashed box are performed iteratively, where a single iteration is equivalent to the discrete passage of a set time-step $\boldsymbol{\delta t}$.

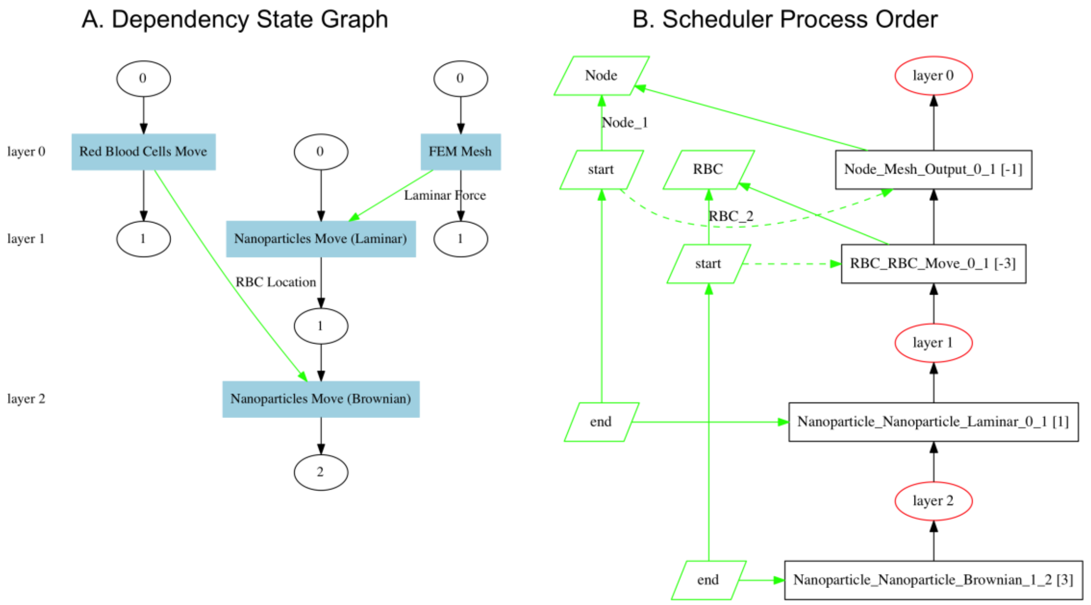

Supplementary Figure 3: FLAME State and Process Order Graphs of the Core Model. The state graph (A) demonstrates the dependency of functions on both previous functions and messages for parallelisation of the core model. The process order graph (B) shows the order in which FLAME prioritises the functions to reduce the lag from using the message passing interface.

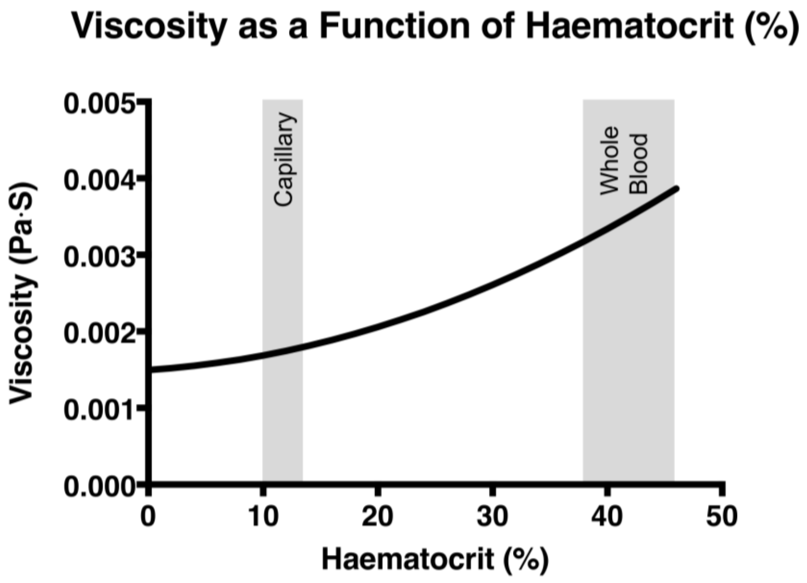

Supplementary Figure 4: The Relationship between Haematocrit and Viscosity. The viscosity used in our models is given by a relationship between haematocrit and viscosity. Capillary and whole blood haematocrits are indicated.

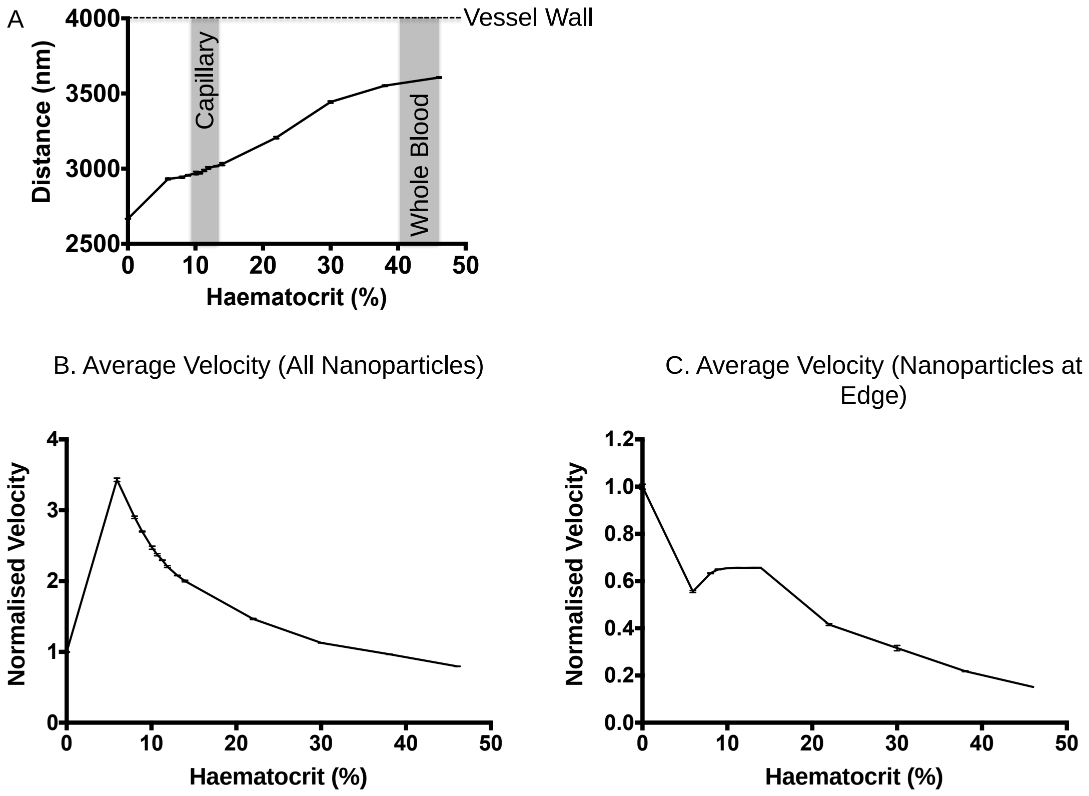

Supplementary Figure 5: The Effect of Haematocrit on Nanoparticle Dispersion and Velocity. The effect of increasing haematocrit on the average position of nanoparticles from the centre of a 4000nm in radius capillary (A). Physiological ranges for capillary and whole blood haematocrits are indicated. The average velocity of, all nanoparticles within the vessel (B) and only nanoparticles within 20nm of the vessel wall (C).

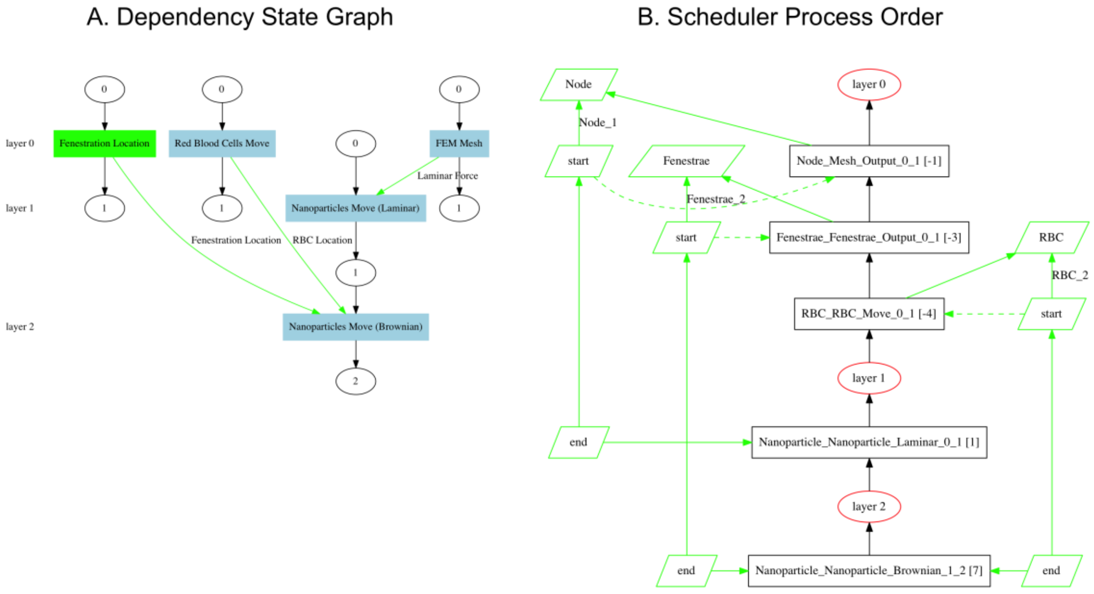

Supplementary Figure 6: FLAME State and Process Order Graphs of the Fenestration Model. The state graph (A) demonstrates the dependency of functions on previous functions and messages for parallelisation of the

fenestration model. The process order graph (B) shows the precise order in which FLAME prioritises the functions to reduce the lag from using the message passing interface.

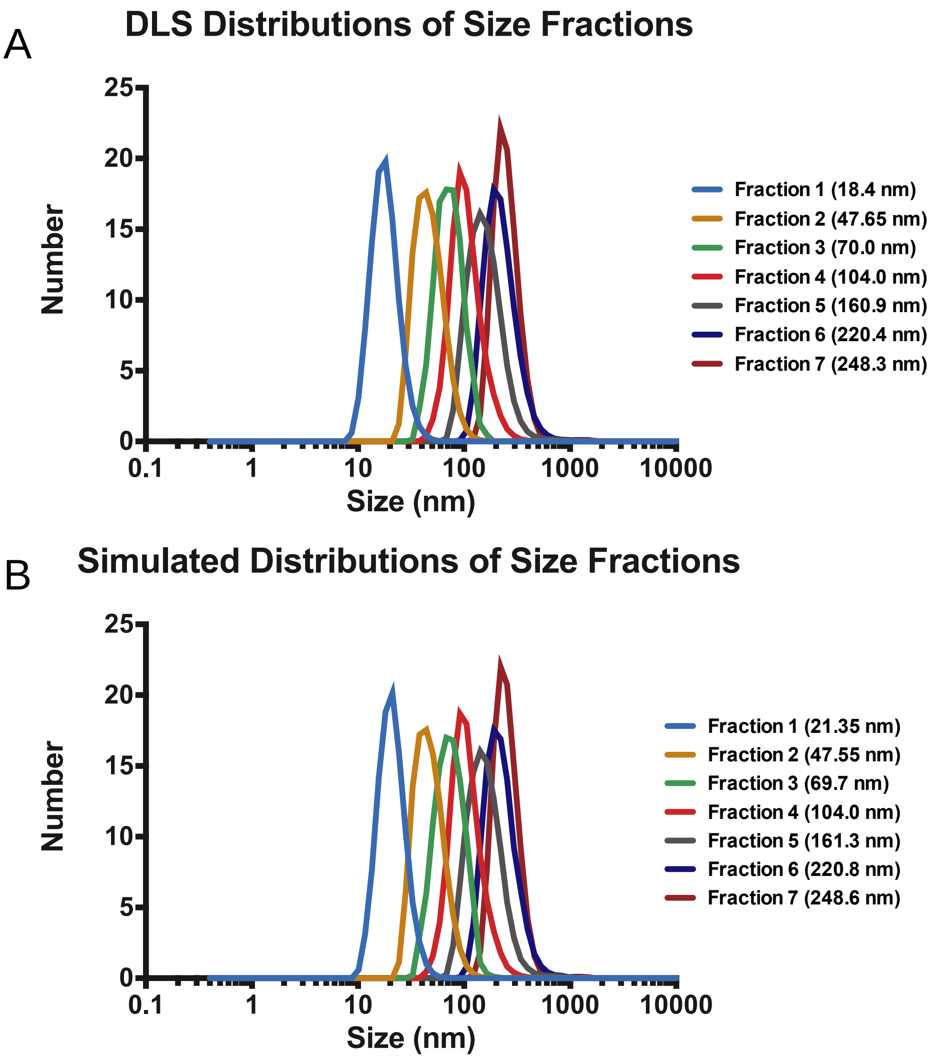

Supplementary Figure 7: Simulating Polydisperse Nanoparticle Distributions from Dynamic Light Scattering (DLS) Data. Size distributions from DLS Zetasizer® software (A) and their corresponding simulated distributions (B) analysed and plotted in the same manner.

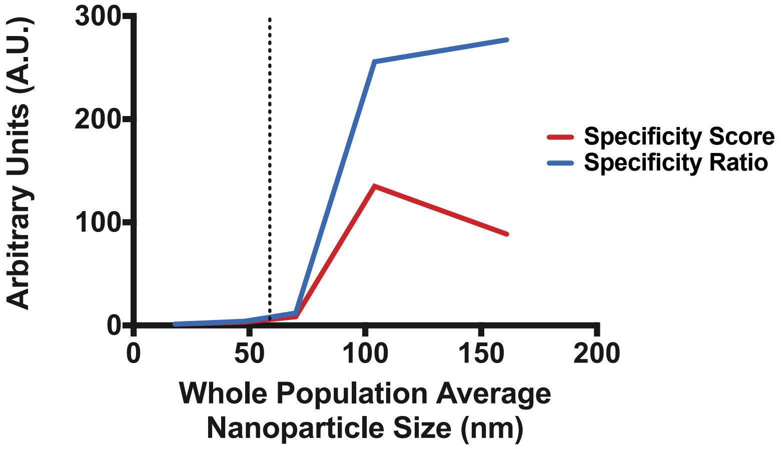

Supplementary Figure 8: Specificity of Different Sized Nanoparticles. The specificity of differently sized nanoparticle populations was evaluated using two measures, the specificity ratio (Equation 9) and specificity score (Equation 10).

1 Chin, L. S. *et al.* FLAME : an approach to the parallelisation of agent-based applications. (2012).
